# Evaluation of Shear Bond Strength in the Repair of Additively and Subtractively Manufactured CAD/CAM Materials Using Bulk-Fill Composites

**DOI:** 10.3390/biomimetics10070433

**Published:** 2025-07-01

**Authors:** Selinsu Öztürk, Ezgi Altuntaş, Ayşe Aslı Şenol, Erkut Kahramanoğlu, Pınar Yılmaz Atalı, Bilge Tarçın, Cafer Türkmen

**Affiliations:** 1 Department of Restorative Dentistry, Faculty of Dentistry, Marmara University, Istanbul 34854, Turkey; asli.tuncer@marmara.edu.tr (A.A.Ş.); pinar.atali@marmara.edu.tr (P.Y.A.); bilge.tarcin@marmara.edu.tr (B.T.); caferturkmen@marmara.edu.tr (C.T.); 2 Department of Restorative Dentistry, Institute of Health Sciences, Marmara University, Istanbul 34865, Turkey; ezgi.altuntas@marun.edu.tr; 3 Department of Prosthodontics, Faculty of Dentistry, Marmara University, Istanbul 34854, Turkey; erkut.kahramanoglu@marmara.edu.tr

**Keywords:** bulk-fill composite, CAD/CAM block, repair bond strength, 3D-printed material

## Abstract

Biomimetic restorative protocols aim to preserve natural tooth structure while enhancing restoration longevity. This in vitro study aimed to evaluate the shear bond strength (SBS) in the repair of additively and subtractively manufactured CAD/CAM materials using bulk-fill resin composites and to assess the effect of thermocycling. Forty rectangular specimens (14.5 × 7 × 3 mm) were prepared from Grandio Blocs (GB, VOCO) and VarseoSmile Crown^Plus^ (VS, BEGO), and thermocycled (5000 cycles, 5–55 °C, 20 s dwell time). All surfaces were roughened with 50 μm Al_2_O_3_. Samples were repaired using VisCalor (VCB, VOCO) and Charisma Bulk Flow One (CBO, Kulzer) composites (*n* = 10 per group) with their respective adhesives. Each group was further divided into immediate and post-thermocycling subgroups. All specimens were tested under shear force until failure, and failure types were examined under a stereomicroscope. Representative samples were examined by SEM to evaluate filler morphology. Statistical analysis was performed with SPSS v23 (*p* < 0.05). No statistically significant differences in SBS were found between groups (*p* > 0.05). Mean SBS values were highest in VS-CBO and lowest in GB-CBO. Cohesive failures were more frequent in immediate groups, while adhesive failures predominated after thermocycling. Bulk-fill composites did not influence the repair bond strength of indirect materials. Thermocycling affected the failure type, though not the SBS values.

## 1. Introduction

The field of computer-aided design and computer-aided manufacturing (CAD/CAM) technology is one of the most rapidly evolving areas in restorative dentistry. The term CAD/CAM refers to various digitally supported techniques, including both additive and subtractive production methods. Subtractive manufacturing involves milling a block of material into the desired shape and is widely used for permanent restorations with resin composites, ceramics, and metals [1,2,3]. Along with various types of ceramic blocks, such as zirconia, reinforced glass, and feldspathic ceramics, new CAD/CAM blocks known as resin-ceramic hybrids have been introduced. These innovative materials combine the benefits of ceramics, such as color stability and durability, with the low wear and high flexural characteristics of resin composites [4,5]. Additive manufacturing, commonly known as three-dimensional (3D) printing, is manufacturing 3D restorations layer by layer [3]. Unlike subtractive manufacturing, 3D printing is more efficient regarding material consumption, with lower costs and a quicker digital workflow. However, to meet the flowability requirements for 3D printing, hybrid ceramic resins typically contain fewer inorganic fillers than hybrid ceramic materials designed for milling [6]. Due to advancements in resin composites and 3D printing technologies, it is now feasible to use 3D-printed CAD/CAM hybrid materials for permanent single-tooth restorations, though the selection of available materials remains limited [7]. Such material developments also support biomimetic restorative concepts by mimicking the mechanical and structural behavior of natural tooth tissues, enabling restorations that preserve function and integrity while minimizing unnecessary tissue loss [8].

Despite the benefits of these materials, fractures can still occur in CAD/CAM materials due to weak interconnections between tooth and restoration, internal stresses, porosity in the material resulting from production, incorrect occlusion, and parafunctional habits. Replacing the restoration may not be the most convenient approach, as it demands further preparation, which can result in unnecessary loss of healthy dental tissue, along with higher costs and loss of time. Direct repair of fractures in indirect restorations with various types of resin composites is now commonly used, providing a more effective treatment option that not only preserves the healthy dental tissue but also allows for quicker and more affordable outcomes [9,10,11]. Such minimally invasive repair strategies align with biomimetic principles by aiming to conserve natural tooth structure while restoring both function and esthetics and help break the cycle of restoration replacements [8]. Several methods are available for repairing indirect restorations using resin composites. These methods involve various surface treatments, adhesive systems, and types of resin composites [12].

Bulk-fill composites present notable benefits in the repair of indirect restorations. Innovations in monomer and filler chemistry have enhanced their properties, providing higher marginal integrity, ease of application, decreased polymerization stresses, and an increased degree of conversion [13]. Bulk-fill composites are generally categorized into two types based on their viscosity as low-viscosity and high-viscosity composites. Low-viscosity composites are also referred to as flowable bulk-fill resin composites [14]. The amount of filler, the matrix formulation, and the manufacturing method determine the viscosity. A lower viscosity is preferable for improved adaptation to fractured surfaces [15]. Charisma Bulk Flow One (Kulzer, Hanau, Germany) is an advanced flowable composite resin material, offering physical characteristics comparable to conventional bulk-fill materials. It is distinguished as the first mono-shade bulk-fill material that eliminates the need for an additional capping layer. By removing the need for shade selection, this material simplifies clinical procedures [16,17]. High-viscosity bulk-fill composites contain more filler in the resin matrix. Increasing the filler ratio improves the mechanical properties of the composite [14]. Temporarily lowering the viscosity of packable resin composites has been highlighted as an effective method to enhance adaptation without affecting the restoration’s mechanical properties. In this context, a bulk-fill resin composite called VisCalor (VOCO, Cuxhaven, Germany) has been specifically developed with viscosity modulation achieved through heating. This material incorporates innovative thermoviscous technology, combining a unique filler surface treatment with a coordinated composite matrix. Compared to conventional resin composites, VisCalor demonstrates significantly improved viscosity reduction, enabling flow characteristics comparable to those of flowable composites, thus facilitating better adaptation and ease of application [18].

For a strong bond between resin-ceramic or resin-composite materials, it is imperative to ensure that both mechanical and chemical adhesion are achieved at the interfaces. [19]. Therefore, surface preparation prior to adhesive application is crucial in the repair procedure to ensure optimal bonding. Various methods, such as sandblasting with alumina (Al_2_O_3_) or silica-coated Al_2_O_3_ powders, diamond burs, or etching with laser or acid (hydrofluoric acid and phosphoric acid), are used to achieve mechanical retention. Chemical bonding is facilitated through the use of adhesive agents [20]. Over the years, adhesive systems used in restorative dentistry have progressed through multiple generations, reflecting significant advancements in formulation and clinical performance. Earlier generations, such as the fourth and fifth, relied on the total-etch technique, which involves separate phosphoric acid etching of both enamel and dentin. While this approach provides strong enamel bonds, it is technique-sensitive and associated with postoperative sensitivity. Later, sixth and seventh generation adhesives introduced self-etch systems that simplified the procedure and reduced sensitivity by incorporating acidic monomers directly into the adhesive, though sometimes at the expense of enamel bond strength. Most recently, eighth generation or so-called universal adhesives were developed, offering the flexibility to be used in total-, self-, or selective-etch modes. These adhesives, including commercially available systems such as Gluma Bond Universal (Kulzer, Hanau, Germany) and Futura Bond U (VOCO, Cuxhaven, Germany), contain functional monomers that promote chemical bonding to a wide range of substrates, including resin composites, zirconia, and CAD/CAM materials. This combination of versatility and simplified technique makes universal adhesives widely used in both direct and indirect restorative procedures. Despite their popularity among clinicians, universal adhesives may exhibit reduced enamel bond strength in self-etch mode and demonstrate variability in performance depending on the substrate. In certain cases, additional surface treatments may still be necessary [21,22,23].

The shear bond test conducted in this study is commonly used in various research to assess the bond strength of repaired materials [24,25]. Replicating the conditions of the oral environment is crucial for predicting the clinical performance of the repaired restoration. As an artificial aging technique, thermal cycling is used to evaluate the effect of temperature fluctuations on shear bond strength (SBS) [26].

While numerous studies have explored the repair of indirect composite restorations, the majority have concentrated on subtractively manufactured CAD/CAM materials, such as milled resins or ceramics. Given the growing clinical use of 3D-printed (additively manufactured) hybrid materials for permanent restorations, there is still limited evidence regarding their repair potential. By integrating contemporary restorative materials with clinically relevant repair procedures, this research provides new insights that may guide optimized repair strategies in digitally driven restorative dentistry.

The aim of this in vitro study was to assess SBS in the repair of additively and subtractively manufactured CAD/CAM materials with bulk-fill resin composites and the impact of the thermocycling procedure. The null hypotheses of the study are:

1: There is no difference between additively and subtractively manufactured CAD/CAM hybrid materials in terms of bond strength.
2: Bond strength is not affected by the type of bulk-fill resin composite used in repair procedures
3: Thermocycling has no effect on the bond strength of the tested materials.

## 2. Materials and Methods

Materials used in the study and their contents are listed in Table 1.

### 2.1. Preparation of Samples

A hybrid CAD/CAM block (Grandio blocs/GB, VOCO, Cuxhaven, Germany) and a 3D-printed material (VarseoSmile Crown^plus^/VS, BEGO, Bremen, Germany) were used in this study. VarseoSmile Crown^plus^ samples were printed in a horizontal orientation with a layer thickness of 50 μm and were supplied by the manufacturer in a cured form as prefabricated blocks [27]. 40 rectangular prism-shaped samples (14.5 mm × 7 mm × 3 mm) were prepared with the Isomet precision cutting device (Buehler, Lake Bluff, IL, USA) under water cooling. Surface standardization of the samples was achieved through 600-grit silicon carbide paper under water cooling for 20 s, and the samples were embedded in self-curing acrylic resin (Imicryl™, Konya, Türkiye) using silicon molds. A thermocycling protocol with 5000 cycles (5–55 °C, dwell time: 20 s) was applied to the samples, corresponding to 6 months of intraoral aging [28].

**Figure 1:**
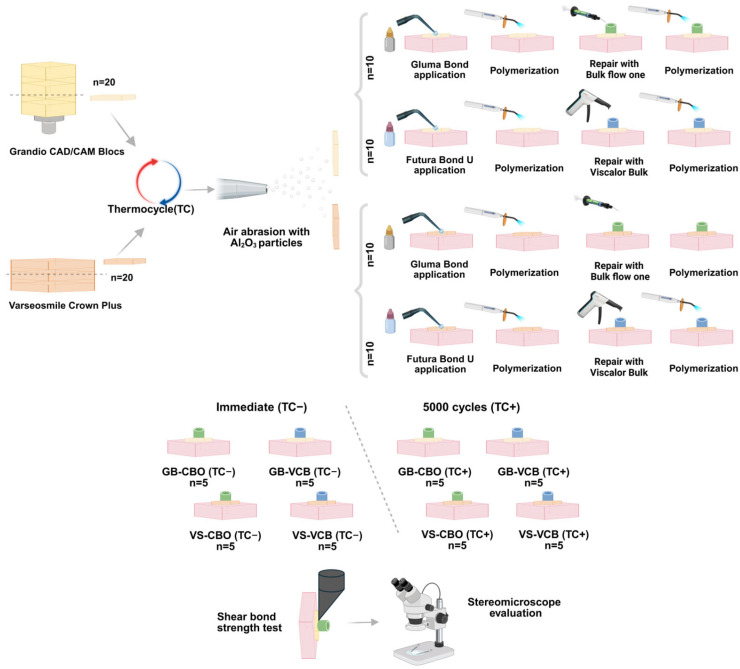
Flowchart of the study protocol and experimental groups.

The surfaces were roughened with 50-μm Al_2_O_3_ particles (10 s, 1.5-bar, and 15-mm distance). After surface preparation, the samples were subjected to ultrasonic cleaning and subsequently air-dried. The samples were then repaired using thermoviscous VisCalor/VCB (VOCO) applied with the VisCalor Dispenser (VOCO) preheating device utilizing near-infrared technology, and flowable Charisma Bulk Flow ONE/CBO (Kulzer) bulk-fill resin composites (*n* = 10/per group) with the materials’ own adhesive systems (Gluma Universal Bond, Kulzer; Futura Bond U, VOCO). Resin composites were placed onto the roughened surface using cylindrical silicone molds (4 × 4 mm) for the repair procedure. Valo a cordless curing device (Ultradent Inc. Products, South Jordan, UT, USA) was used in standard mode for polymerization (1000 mW/cm^2^ for 20 s), following the instructions of the manufacturer. The samples were divided into two subgroups, and the thermocycling procedure was repeated in only one group to simulate the aging of the repaired restoration (5000 cycles; 5–55° C, dwell time: 20 s). The other group was not subjected to thermocycling and was described as the immediate group (Figure 1).

### 2.2. Shear Bond Strength Test

Samples from all groups were loaded in a Shimadzu AGS-X Universal testing device (Shimadzu Corporation, Kyoto, Japan) with a crosshead speed of 1 mm/min. Failure load was recorded for each sample and calculated using the following formula:

(1) $$Shear\;Bond\;Strength\;\left(MPa\right)=\frac{load\;at\;failure\;(N)}{bonded\;surface\;area\;(mm2)}$$

### 2.3. Failure Type Evaluation

All samples were examined under a Leica MZ 75 stereomicroscope (Leica Microsystems, Heerbrugg, Switzerland) under 20× magnification by the same operator to determine the failure mode. Failures were classified as: adhesive (failure at the composite/indirect restoration material interface), cohesive (failure within the composite resin or indirect restoration material), and mixed (including both types of failures).

### 2.4. Scanning Electron Microscopy (SEM) Evaluation

Additional samples (three samples per group) from VS, GB, VCB, and CBO were prepared for evaluation under SEM (Evo™ MA10; Zeiss, Oberkochen, Germany) operated at an accelerating voltage of 10.00 kV. The samples were rinsed with ethanol solution to ensure dehydration before SEM evaluation, and then the imaging surface was sputter-coated with gold-palladium. The internal structure and filler morphology of bulk-fill composite cylinders and CAD/CAM block sections were examined at ×1000 magnification.

### 2.5. Statistical Analysis

Sample size determination was performed using G* Power v3.1.9.7 software (Düsseldorf, Germany) to determine the minimum sample size. Results indicated the required sample size to achieve 80% power with α = 0.05 was N = 40 (5 per group) [29,30].

Data were analyzed with PSS V23 (IBM, Armonk, NY, USA). Normal distribution was analyzed by the Shapiro–Wilk test. Homogeneity of variances was assessed using Levene’s test. Three-way ANOVA was used to compare SBS values according to indirect material, composite, and time. Fisher’s Exact and Fisher Freeman Halton tests were used to compare the failure type according to the groups. Categorical variables were summarized as frequencies (percentages), while quantitative variables were expressed as mean ± standard deviation. The significance level was set at *p* < 0.05.

## 3. Results

### 3.1. Shear Bond Strength (SBS)

There is no statistically significant difference between the mean SBS values according to the type of composite and indirect restorative material (*p* = 0.628, *p* = 0.104, respectively) (Table 2). The mean SBS value for the Bulk Flow One resin composite was 11.84 MPa, whereas the VisCalor Bulk was 11.12 MPa. The mean SBS value for the VarseoSmile Crown^plus^ was 12.73 MPa, while the Grandio Blocs had a mean of 10.24 MPa (Table 3).

There is no statistically significant difference between the mean SBS values according to the composite resin and indirect restoration material interaction (*p* = 0.322). The mean SBS values in decreasing order were as follows (in MPa): VS-CBO (13.84 ± 4.90) > VS-VCB (11.61 ± 6.03) > GB-VCB (10.62 ± 2.67) > GB-CBO (9.85 ± 4.41) (Table 2 and Table 3).

No statistically significant difference was observed between the mean SBS values according to the interaction of aging procedure, type of composite resin, and indirect restorative material (*p* = 0.621) (Table 2).

No statistically significant differences were detected between the total mean SBS values as an effect of the thermocycling (*p > 0.05*). The total mean SBS value was 10.87 ± 3.51 MPa in the immediate group, whereas in the test group it was determined as 12.09 ± 5.73 MPa following thermocycling (Table 3).

### 3.2. Failure Type

**Figure 2:**
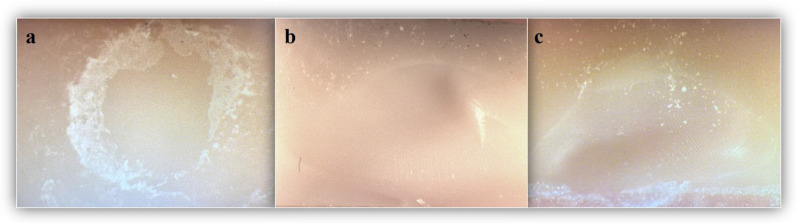
Failure types: (**a**) adhesive type; (**b**) cohesive type; and (**c**) mixed type.

Except for the repair of VS with CBO (*p* = 0.167), all other repair combinations demonstrated a statistically significant difference in the distribution of failure types—adhesive, cohesive, and mixed— between immediate and thermocycled groups (*p* < 0.05). Regardless of the composite or material type, cohesive failures accounted for 75% of the failures in the immediate groups, whereas adhesive failures predominated in the thermocycled groups, reaching 80% (Table 4, Figure 2).

### 3.3. Scanning Electron Microscopy (SEM)

**Figure 3:**
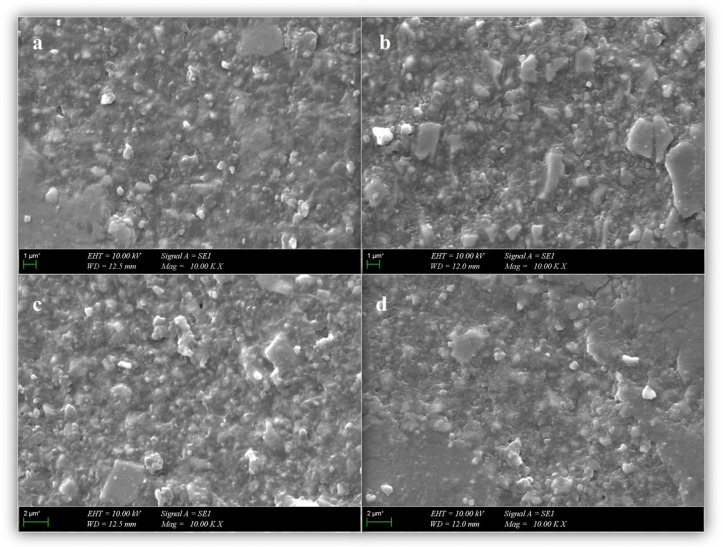
SEM evaluation of CAD/CAM materials and bulk-fill resin composites: (**a**) Varseosmile Crown^plus^; (**b**) Grandio Blocs; (**c**) Charisma Bulk Flow One; and (**d**) VisCalor Bulk.

SEM micrographs of the representative samples are shown in Figure 3. SEM micrographs suggested noticeable variations in the microstructure of the additively and subtractively manufactured indirect restorative materials. VarseoSmile Crown^plus^ appeared to exhibit a relatively homogeneous resin matrix with sparsely distributed, uniformly small filler particles, which may reflect a lower inorganic filler content. In comparison, Grandio Blocs tended to show a more densely packed filler arrangement with irregularly shaped and variably sized particles. Similarly, SEM micrographs of the direct restorative materials, VisCalor Bulk and Charisma Bulk Flow One, demonstrated nanohybrid structures composed of both nano- and micro-sized inorganic fillers. In both composites, barium aluminum glass and silicon dioxide were observed as the main filler components. VisCalor Bulk showed a relatively uniform and compact filler distribution, while Charisma Bulk Flow One presented visibly larger filler particles, as stated by the manufacturer.

## 4. Discussion

Although there are many studies on indirect composite repair, most focus on subtractively manufactured CAD/CAM materials. With the growing use of 3D-printed (additively manufactured) CAD/CAM hybrid materials for permanent single-tooth restorations, their repair has become increasingly relevant in clinical practice. In the present study, VS, which contains 30–50% inorganic fillers, was used as a 3D printing material, while GB, with 86% inorganic filler content, was used as a subtractively manufactured CAD/CAM material. Due to the flowability requirements of the 3D printing process, VS contains a lower number of inorganic fillers. This compositional difference was also reflected in the SEM images (Figure 3), where VS revealed a more uniform resin matrix with sparsely distributed and finer filler particles, in contrast to the compact filler configuration with non-uniform shapes observed in GB (Figure 3).

The mechanical properties and bonding performance of 3D-printed restorations are affected by the material composition, as well as the printing orientation and process, which influence the internal structure and surface morphology of the printed restoration [31,32]. Comparative studies on different printing orientations, namely horizontal, vertical, and angled, have shown that the horizontal orientation generally results in superior mechanical performance [33,34]. However, a study evaluating the shear bond strength of 3D-printed materials repaired with flowable resin composites reported that the printing orientation did not significantly affect the bond strength. Notably, the use of flowable composites yielded high bond strength values, highlighting their favorable performance in such repair protocols [32]. In this study, the 3D-printed material samples were fabricated in a horizontal orientation, as recommended by the manufacturer, aligning the bonding surface parallel to the layering direction. This orientation may have favorably influenced the surface roughness and adhesive penetration following sandblasting.

Nevertheless, no significant difference in SBS was observed between the additively and subtractively manufactured indirect materials. Therefore, the first null hypothesis (There is no difference between additively and subtractively manufactured CAD/CAM hybrid materials in terms of bond strength) is accepted.

Intraoral repair techniques are widely preferred in routine clinical applications due to their minimally invasive nature and cost-effectiveness [28]. With current progress in their properties, composite resins have become a common choice for repairing indirect hybrid material restorations. The type of resin material used in intraoral repair procedures is a factor that impacts the bond strength between the composite resin and the restoration. Both the filler composition and particle size of the composite resin can significantly impact the success of the bond strength [9]. This study aimed to reduce the risk of material failure by eliminating layering and enhancing the conversion degree through bulk-fill composites, VCB, and CBO as repair materials. VCB is a packable resin material with 83% inorganic filler content and a particle size range of 0.02 to 1.2 µm, which becomes highly flowable when heated to 68 °C [35]. On the other hand, CBO is a flowable bulk-fill composite with 65% inorganic filler content and a particle size range of 0.02 to 5 μm [16]. Ultrastructural observations by SEM indicated that these bulk-fill resin composites exhibited a homogeneous nanohybrid structure, characterized by relatively well-dispersed filler particles that may contribute to their favorable mechanical behavior (Figure 3).

In the present study, no statistically significant difference was found in the mean SBS values based on the type of bulk-fill composites. For this reason, the second null hypothesis (Bond strength is not affected by the type of bulk-fill resin composite used in repair procedures) is accepted. According to previous studies, a minimum bond strength of 6–8 MPa is necessary for successful intraoral repairs [16,36], and other studies have reported 10 MPa as the critical SBS value [9,37], while ISO standard 10477 specifies that at least 5 MPa is required for materials to achieve acceptable retention [38]. However, it may not be suitable to compare SBS values obtained from different testing methods. In this study, the lowest SBS value, obtained in GB repaired with CBO (thermocycled), was 8.57 MPa, and the average bond strength values for all tested materials exceeded the 5 MPa threshold, indicating clinically successful intraoral repairs. In the present study, the flowable structure of CBO, which does not require additional capping material, and the viscosity reduction of VCB through heat application are considered to enhance their adaptation to the repair surfaces. As also reported by Şenol et al., the repair of Grandio blocs with VisCalor achieved SBS values exceeding the limits considered clinically acceptable [35]. Loumprinis et al. demonstrated that heating VisCalor to 54 °C resulted in the greatest reduction in viscosity, achieving a level comparable to that of flowable resin composites, while no significant increase in stickiness was observed [18]. Additionally, another study reported a significant decrease in internal porosity (− 65.4%) where the VisCalor injection technique was employed in contrast to the conventional resin composite placement method [39]. Therefore, the combination of CAD/CAM materials with bulk-fill composite resins is thought to optimize bond strength by reinforcing their respective advantages, including simplicity of use and application, reduced polymerization stresses, increased marginal integrity, and conversion degree.

Both chemical and mechanical treatments play a key role in establishing a durable and high-quality repair interface [40]. Surface treatment during repair varies based on the composition of restorative materials, including air abrasion with Al_2_O_3_ particles and silica-coated Al_2_O_3_ particles, diamond burs, acid, or laser etching. However, the optimal surface treatment approach remains a subject of discussion within the literature. Studies have demonstrated that air abrasion with Al_2_O_3_ particles significantly enhances repair strength with the advantages of reduced heat, vibration, and noise [24,41,42]. The Al_2_O_3_ particles create surface irregularities that promote micromechanical interlocking between the surface to be repaired and the resin material, improving the bond strength [43]. Chemical treatment may include the application of silane and/or adhesive by the clinician [44]. Certain universal adhesives are formulated with silane, which improves adhesion between ceramic substrates and composite resins. The presence of silane in these adhesives enables chemical bonding during the cementation or repair of ceramic and indirect composite restorations without requiring a separate priming agent. Incorporating bi-functional silane monomers into universal bonding agents simplifies the procedure by reducing the number of steps, allowing these formulations to function as both dentin and enamel adhesives [15]. Regarding their silane content, the universal adhesives in the present study (Gluma Bond and Futura Bond U) can serve as active adhesives, eliminating the need for an additional silane primer when used as repair procedure materials.

Failure of indirect restorative materials typically occurs over time in the oral environment. Even though a gold standard for the aging of these materials has not been established, thermal aging is the most employed method to simulate the thermal fluctuations experienced under oral conditions. The number of thermal cycles used in aging protocols varies among studies. Most studies employ 10,000 cycles; however, some studies use fewer cycles [12]. In this study, the samples underwent 5000 cycles of thermal aging before applying the repair procedure. The thermocycling process was repeated for half of the samples of each group to evaluate the effects of aging after repair. Repair bond strength was not affected by thermocycling in all tested indirect hybrid materials. These findings are consistent with those reported by Mao et al. [6] and Graf et al. [45], while other studies indicate that thermocycling negatively affects retention [28,35]. Consequently, the third null hypothesis of the study (Thermocycling has no effect on the bond strength of the tested materials) is accepted.

Failure type assessment is a critical aspect of bond strength research. Identifying the types of failures helps evaluate the clinical performance of CAD-CAM materials and the repair methods applied. In this study, failure types for each group were examined under a stereomicroscope, and surface fracture patterns were categorized as adhesive, cohesive, or mixed. Cohesive-type fractures were predominantly observed in the immediate groups, whereas adhesive-type fractures were more frequent in the thermocycled groups. This finding is in agreement with a previous study, which similarly reported cohesive fractures in fresh composites, while adhesive failures were predominantly observed at the bonding interface in aged composite specimens [46]. It may be inferred that the adhesive interface is more prone to degradation than the cohesive phase in thermally aged samples [47].

A variety of tests can be employed to assess bond strength, including tensile and micro tensile, shear and micro shear bond strength, and pull-out tests. The macro shear bond strength test, which is a traditional loading method with a knife-edge chisel, was preferred for evaluating samples in this study due to its simplicity, highly reproducible test procedures, and low risk of failure in the pre-test phase [28,48]. Nevertheless, the non-uniform distribution of stress in the adhesive area should be considered.

While this study provides valuable insights, such as the use of both subtractively and additively manufactured CAD/CAM restorative materials, the application of standardized bonding protocols, and the use of currently available bulk-fill composites, several limitations should also be acknowledged. Only a single 3D-printed CAD/CAM material was used, which may limit the applicability of the findings to other additively manufactured restorative materials. Incorporating a wider range of 3D-printed CAD/CAM materials into similar repair protocols may provide broader comparative data and enhance understanding of material-specific bonding behavior. As only bulk-fill composites were utilized in the repair procedures, the findings are most relevant to this material category; exploring other composite types in future research could provide broader comparative insights. Given that this study employed a single surface treatment protocol, future investigations may benefit from comparing multiple surface preparation techniques. Future research could incorporate micro shear and micro tensile tests, considering their respective advantages and limitations. Additionally, different aging procedures and durations should be examined to evaluate their effect on bond strength. The in vitro nature of this study limits the ability to replicate the complex and dynamic conditions of the oral environment. Therefore, further in vivo investigations are necessary to validate the clinical relevance of the results and the tested materials.

## 5. Conclusions

Within the limitations of this in vitro study, the bulk-fill resin composites used in this study did not affect the repair procedure of Grandio Blocs and VarseoSmile Crown^plus^. Although thermocycling did not affect the repair bond strength, it had a decisive effect on the failure type. All tested materials demonstrated clinically acceptable bond strength values for a successful intraoral repair in accordance with the ISO standards.

## Figures and Tables

**Table 1:** Materials and their contents used in the study.

Types of Materials	Material Name	Code	Manufacturer	Contents
Indirect Restorative Materials	Grandio Blocs	GB	VOCO GmbH, Cuxhaven, Germany	Nanohybrid composite CAD/CAM block Fillers: Barium aluminum borosilicate glass, SiO_2_, filler loading 86 wt%
				Matrix: polymethacrylate, stabilizers, pigments
	VarseoSmile Crown^plus^	VS	BEGO, Bremen, Germany	Hybrid 3D-Printed material Fillers: Ceramic-filled (30–50 wt% inorganic fillers; silanized dental glass),
				Matrix: Methylbenzoylformate, diphenyl (2,4,6-trimethylbenzoyl), Phosphine oxide hybrid material
Bulk-fill Resin Composites	VisCalor Bulk	VCB	VOCO GmbH, Cuxhaven, Germany	Nanohybrid Resin Composite Fillers: Barium aluminum borosilicate glass, SiO_2_, filler loading 83 wt%
				Matrix: BisGMA, TCDDMA, initiators, stabilizers, color pigments,
	Charisma Bulk Flow One	CBO	Kulzer, Hanau, Germany	Nanohybrid Resin Composite Fillers: Barium aluminum fluorosilicate glass, SiO_2_, ytterbium fluoride, filler loading 65 wt%
				Matrix: UDMA, EBADMA, photoinitiators
Universal Adhesives	Gluma Universal Bond	GU	Kulzer, Hanau, Germany	Eighth Generation Adhesive Resin Fillers
				Matrix: MDP phosphate monomers, 4-META, dimethacrylate resins, acetone, initiators
				Silane
	FuturaBond U	FU	VOCO GmbH, Cuxhaven, Germany	Eighth Generation Adhesive Resin Fillers: Functionalized SiO_2_
				Matrix: Ethanol, Bis-GMA, HEMA, H_2_O, HEDMA, methacrylate phosphoric acid ester, methacrylate-modified polyacid, UDMA, initiators, stabilizers
				Silane

Abbreviations: Bis-GMA: bisphenol A-glycidyl methacrylate, UDMA: urethane dimethacrylate, HEMA: 2-hydroxylethyl methacrylate, HEDMA: 2-hydroxyethyl dimethylamine, TCDDMA: tricyclodecane dimethanol dimethacrylate, EBADMA: ethoxylated bisphenol A dimethacrylate, SiO_2_: silicon dioxide.

**Table 2:** Comparison of SBS values according to the type of resin composite, indirect material, and aging procedure.

	F	*p-Value*	Partial Eta-Squared
Resin Composite	0.24	0.628	0.007
Indirect Material	2.806	0.104	0.081
Aging	0.668	0.42	0.02
Resin Composite * Indirect Material	1.012	0.322	0.031
Resin Composite * Aging Procedure	0.312	0.58	0.01
Indirect Material * Aging Procedure	2.186	0.149	0.064
Resin Composite * Indirect Material * Aging Procedure	0.25	0.621	0.008

F: variance analysis, R^2^ = 0.189, Corrected R^2^ = 0.012.

**Table 3:** Descriptive statistics of SBS values by composite, material, and aging procedure.

Indirect Material	Aging Procedure	Resin Composite		Total
		Charisma Bulk Flow One	VisCalor Bulk	
VarseoSmile Crown^plus^	Immediate	12.17 ± 2.68	9.86 ± 1.72	11.02 ± 2.45
	TC	15.50 ± 6.31	13.36 ± 8.44	14.43 ± 7.12
	Main effect ^1^	13.84 ± 4.90	11.61 ± 6.03	12.73 ± 5.47
Grandio Blocs	Immediate	11.13 ± 5.55	10.32 ± 3.71	10.73 ± 4.47
	TC	8.57 ± 2.97	10.91 ± 1.43	9.74 ± 2.52
	Main effect ^1^	9.85 ± 4.41	10.62 ± 2.67	10.24 ± 3.57
Total	Immediate	11.65 ± 4.15	10.09 ± 2.74	10.87 ± 3.51
	TC	12.04 ± 5.91	12.14 ± 5.85	12.09 ± 5.73
	Main effect ^1^	11.84 ± 4.97	11.12 ± 4.57	11.48 ± 4.73

All values are presented as mean ± standard deviation. TC: thermocycle; main effect ^1^: composite main effect regardless of aging. For statistical comparisons and corresponding *p*-values, please refer to Table 2.

**Table 4:** Comparison (%) of failure types according to indirect material, aging procedure, and resin composite.

Indirect Material	Resin Composite	Failure Type	Aging Procedure		Total	*p-Value*
			Immediate	TC		
Varseosmile Crown^Plus^	Charisma Bulk Flow One	Adhesive	0 (0)	2 (40)	2 (20)	0.167 **
		Cohesive	3 (60)	0 (0)	3 (30)	
		Mixed	2 (40)	3 (60)	5 (50)	
	VisCalor Bulk	Adhesive	0 (0) ^a^	5 (100) ^b^	5 (50)	0.008 **
		Cohesive	4 (80) ^a^	0 (0) ^b^	4 (40)	
		Mixed	1 (20)	0 (0)	1 (10)	
	Main effect ^2^	Adhesive	0 (0) ^a^	7 (70) ^b^	7 (35)	<0.001 **
		Cohesive	7 (70) ^a^	0 (0) ^b^	7 (35)	
		Mixed	3 (30)	3 (30)	6 (30)	
Grandio Blocs	Charisma Bulk Flow One	Adhesive	0 (0) ^a^	4 (80) ^b^	4 (40)	0.016 **
		Cohesive	4 (80) ^a^	0 (0) ^b^	4 (40)	
		Mixed	1 (20)	1 (20)	2 (20)	
	VisCalor Bulk	Adhesive	1 (20) ^a^	5 (100) ^b^	6 (60)	0.048 *
		Cohesive	4 (80) ^a^	0 (0) ^b^	4 (40)	
	Main effect ^2^	Adhesive	1 (10) ^a^	9 (90) ^b^	10 (50)	<0.001 **
		Cohesive	8 (80) ^a^	0 (0) ^b^	8 (40)	
		Mixed	1 (10)	1 (10)	2 (10)	
Total	Charisma Bulk Flow One	Adhesive	0 (0) ^a^	6 (60) ^b^	6 (30)	0.001 **
		Cohesive	7 (70) ^a^	0 (0) ^b^	7 (35)	
		Mixed	3 (30)	4 (40)	7 (35)	
	VisCalor Bulk	Adhesive	1 (10) ^a^	10 (100) ^b^	11 (55)	<0.001 **
		Cohesive	8 (80) ^a^	0 (0) ^b^	8 (40)	
		Mixed	1 (10)	0 (0)	1 (5)	
	Main effect ^2^	Adhesive	1 (5) ^a^	16 (80) ^b^	17 (42,5)	<0.001 **
		Cohesive	15 (75) ^a^	0 (0) ^b^	15 (37,5)	
		Mixed	4 (20)	4 (20)	8 (20)	

* Fisher’s Exact test, ** Fisher Freeman Halton test. The same letters indicate no statistical difference between the groups. TC: thermocycle; main effect ^2^: aging main effect regardless of the composite.

## Data Availability

The data presented in this study are available on request from the corresponding author.
